# Infant mortality in the municipality of São Paulo: trend and social inequality (2006–2019)

**DOI:** 10.11606/s1518-8787.2023057004791

**Published:** 2023-10-30

**Authors:** Katia Cristina Bassichetto, Margarida Maria de Azevedo Tenório Lira, Edige Felipe de Sousa Santos, Ivan Arroyave, Samantha Hasegawa Farias, Marilisa Berti de Azevedo Barros

**Affiliations:** I Faculdade de Ciências Médicas Santa Casa de São Paulo Departamento de Saúde Coletiva São Paulo SP Brazil Faculdade de Ciências Médicas da Santa Casa de São Paulo . Departamento de Saúde Coletiva . São Paulo , SP , Brazil .; II Independent researcher São Paulo SP Brazil Independent researcher . São Paulo , SP , Brazil .; III Universidade de São Paulo Faculdade de Saúde Pública Departamento de Epidemiologia São Paulo SP Brazil Universidade de São Paulo . Faculdade de Saúde Pública da USP. Departamento de Epidemiologia . São Paulo , SP , Brazil .; IV Universidad de Antioquia Facultad Nacional de Salud Pública Medellín Colombia Universidad de Antioquia . Facultad Nacional de Salud Pública . Medellín , Colombia .; V Universidade Estadual de Campinas Faculdade de Ciências Médicas Departamento de Saúde Coletiva Campinas SP Brazil Universidade Estadual de Campinas . Faculdade de Ciências Médicas . Departamento de Saúde Coletiva , Campinas , SP , Brazil .

**Keywords:** Infant mortality, Time Factors, Socioeconomic Factors, Social Vulnerability

## Abstract

**OBJECTIVE:**

Considering the published evidence on the impact of recent economic crises and the implementation of fiscal austerity policies in Brazil on various health indicators, this study aims to analyze how the trend and socio-spatial inequality of infant mortality behaved in the municipality of São Paulo from 2006 to 2019.

**METHODS:**

This is an ecological study with a temporal trend analysis that was developed in municipality of São Paulo, using three residence area strata differentiated according to their social vulnerability following the 2010 São Paulo Social Vulnerability Index. Infant mortality rate, as well as neonatal, and post-neonatal mortality rates, were calculated for each social vulnerability stratum, each year in the period, and for the first and last three triennia. Temporal trends were analyzed by the Prais-Winsten regression model and inequality magnitude, by rate ratios.

**RESULTS:**

We found a decline in infant mortality rate and its components from 2006 to 2015, greater in the stratum with low social vulnerability and in the post-neonatal period when compared to the neonatal one. This decline ended in 2015, stagnating in the next period (2016–2019). Our analysis of infant mortality inequality across social vulnerability stratum showed a significant increase from the initial to the final triennia in the analyzed period; rate ratios increased from 1.36 to 1.48 in the high stratum (compared to the low social vulnerability stratum), and from 1.19 to 1.32 between the medium and low social vulnerability strata.

**CONCLUSIONS:**

The observed stagnation of infant mortality rate decline in 2015 and the increase in socio-spatial inequality point to the urgent need to reformulate current public policies to reverse this situation and reduce inequalities in the risk of infant death.

## INTRODUCTION

Data from the World Health Organization show that infant mortality rates (IMR) declined by about 50% from 2000 to 2018, but unequally across regions and countries ^1^ . This panorama also occurred in the Americas, whose IMR fell by 55% from 1995 to 2017, but with great variation between countries ^2^ .

The literature has shown that accelerated income concentration, economic crises, and fiscal austerity policies have negatively affected several health indicators ^3^ and infant mortality in several countries of the world ^4 - 6^ .

In Brazil, these same processes have also worsened the trends of some health indicators (risk and protective factors for chronic non-communicable diseases, premature mortality rates due to them, and that of infants and mothers), increasing social inequality among social segments of these populations ^7^ . Regarding infant mortality, a Brazilian study found, in 2016 and 2017, a deceleration of the downward trend observed up to 2015 ^8^ and two other studies found an increase in IMR in 2016, after the decline up to 2015 ^9 , 10^ . 2016 deepened the economic and political crisis in Brazil, unevenly affecting its overall population and that of the municipality of São Paulo (MSP), increasing unemployment rates and decreasing income, especially among the most vulnerable population ^11^ .

In view of this situation and considering the relevance of IMR (a sensitive indicator of local living and health conditions ^12 , 13^ ) and the absence of recent studies on the trend of rates and social inequalities of this indicator in MSP, our study aims to analyze rate trend and the magnitude of socio-spatial inequalities of infant mortality in this municipality from 2006 to 2019.

## METHODS

### Type of study

This is an ecological study with a time series design that uses MSP data on deaths in children under one year of age and live births in areas with different levels of social vulnerability, fron 2006 to 2019.

Data on deaths and live births were collected by place of residence and corresponded to the period from January 1, 2006 to December 31, 2019, as the latter was considered the most recent year with available consolidated data. We chose to use 2006 as the first year of the series as it was only from this date that the Secretaria Municipal de Saúde de São Paulo (São Paulo Municipal Health Secretariat SMS-SP) began to feedback records of the Ministry of Health database, incorporating the events that occurred in other municipalities into its municipal database.

Our databases come from the *Sistema de Informação de Mortalidade* (SIM –Mortality Information Systems) and that on *Sistema de Informações sobre Nascidos Vivos* (SINASC – Live Birth Information System). The population residing in MSP was obtained from the SMS-SP TabNet, with projections by the SEADE Foundation based on data from the 2010 Census ^14^ .

### Analysis of Socio-Spatial Inequalities

Socio-spatial inequalities were analyzed based on the São Paulo Social Vulnerability Index (SPSVI) by the SEADE Foundation, which used socioeconomic and demographic indicators to classify the census tracts of the municipalities in the state of São Paulo into six social vulnerability groups: lowest, very low, low, medium, high, and very high. This indicator enables the identification and spatial location of the areas housing the segments exposed to different degrees of social vulnerability ^15^ .

**Figure 1:**
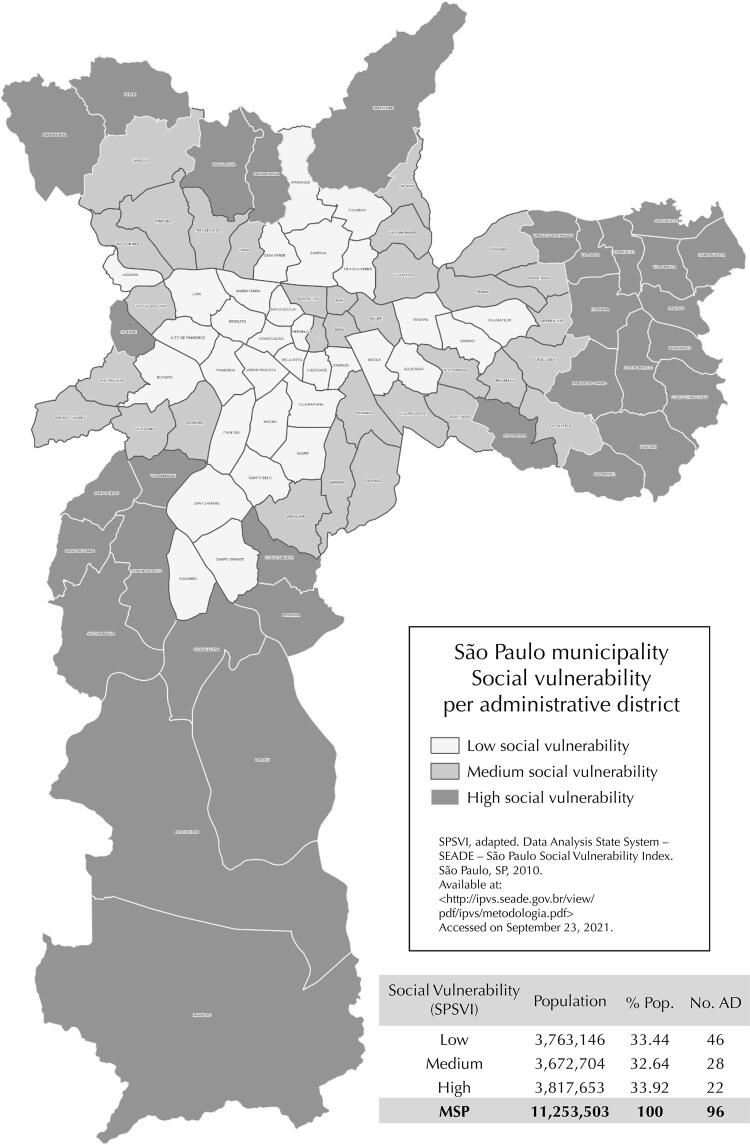
Classification of the administrative districts (DA) of the municipality of São Paulo according to social vulnerability, adapted from the São Paulo Social Vulnerability Index (SEADE Foundation, 2013).

MSP: São Paulo municipality; AD: administrative district.

Source: adapted from the São Paulo Social Vulnerability Index (SPSVI), SEADE Foundation, 2013.

To classify the MSP Social Vulnerability Strata, each of its 96 Administrative Districts (AD) received a vulnerability score based on the percentage of census tracts classified in each SPSVI group. Then, AD were ordered from lowest to highest according to these scores and classified into three social vulnerability strata (low, medium, and high) ( Figure 1 ), containing about one third of the population in the municipality. The low social vulnerability stratum included 46 AD; the medium stratum, 28, and the high vulnerability stratum, 22.

### Statistical Analyses

IMR (< 1 year of age) and neonatal (0–27 days) and post-neonatal (28 days to < 1 year) mortality rates per 1,000 live births (LB) were calculated for the three social vulnerability strata for each year in the studied period (2006 to 2019).

The Prais-Winsten regression model was used to analyze trends. Infant, neonatal, and post-neonatal mortality trends were analyzed for the whole period (2006–2019) and separately from 2006 to 2015 and from 2016 to 2019 since we found the lowest IMR in 2015. For the time series, the rate logarithm was considered as the dependent variable and the years in the historical series as the independent variable. Serial autocorrelation was checked using the Durbin-Watson test, whereas rate annual percentage variation (APV), regression coefficients (β), respective 95% confidence intervals (95%CI), and p-values (p) were calculated considering a 95% significance level ^16^ .

To analyze the magnitude of infant mortality inequality, the extreme years in the series were grouped into three-year periods (2006–2008 and 2017–2019) and the rate ratios (RT) between high and medium social vulnerability areas were calculated in relation to those with low vulnerability and used as a reference category. This procedure was performed to provide greater rate stability for our analyses.

To evaluate whether the RT between the areas with high and medium social vulnerability, compared to those with low vulnerability, differed between the first and the last triennium, the right-tailed Student’s t-test was used for independent samples. A p-value < 0.05 was considered for statistical decision-making.

Data tabulation, descriptive analysis, and graphs were performed in TabWin (tabulator for windows, developed by DATASUS) and Microsoft Office Excel 2010. All analyses were carried out using Stata 15.0 ^®^ (Stata Corp, LP).

In this study, databases of deaths and live births without identification available electronically and aggregated by the SMS-SP were used, thus dispensing with submitting this study to the Research Ethics Committee. This research was conducted in accordance with the Resolution of the National Health Council no. 466 of December 12, 2012 ^17^ .

## RESULTS

**Figure 2:**
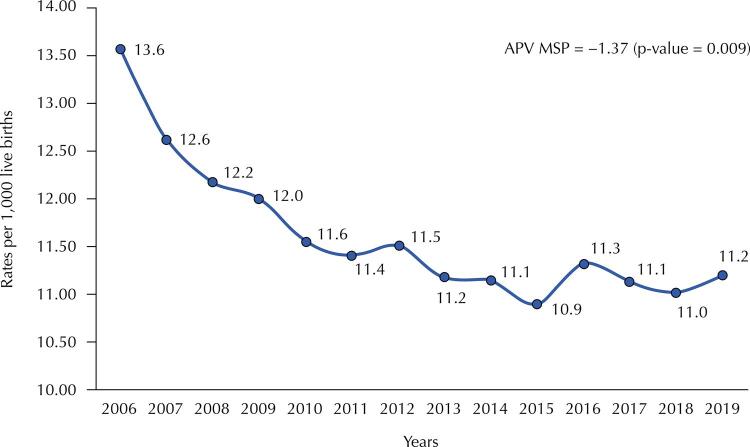
Infant mortality trend. Municipality of São Paulo, 2006–2019.

APV: annual percentage variation; MSP: municipality of São Paulo.

MSP suffered 27,808 deaths in children under 1 year of age and had 2,394,375 LB, from 2006 to 2019. Figure 2 shows that infant mortality declined from 13.6 deaths/1,000 LB in 2006 to 10.9/1,000 LB in 2015. This decline ended and the rate had a value of 11.2/1,000 LB in 2019.

We observed that about 65% of infant deaths occurred in the neonatal component for all strata of social vulnerability in the first and last analyzed triennia ( Table 1 ).

**Table 1:** Absolute (N) and relative values (% and rates) of infant deaths and their components, according to social vulnerability strata. Municipality of São Paulo, 2006–2008.

Social vulnerability strata	2006–2008			2017–2019		
	
	n	%	Rate	n	%	Rate
Low						
Neonatal	929	65.2	6.88	743	69.6	5.9
Post-neonatal	495	34.8	3.66	325	30.4	2.58
Infant	1,424	100	10.53	1,068	100	8.48
Medium						
Neonatal	1,427	64.8	8.14	1,234	65.9	7.42
Post-neonatal	776	35.2	4.43	637	34.1	3.83
Infant	2,203	100	12.57	1,871	100	11.24
High						
Neonatal	1,840	65.6	9.42	1,669	67.7	8.51
Post-neonatal	964	34.4	4.94	797	32.3	4.06
Infant	2,804	100	14.35	2,466	100	12.58

Our analysis of the IMR trend for the whole period ( Table 2 ) shows a significant decline of −1.40% a year but this decrease actually occurred from 2006 to 2015, with an APV of −2.25% a year (p = 0.001), whereas no significant decline occurred from 2016 and 2019 (APV = −0.44; p = 0.481).

**Table 2:** Trends in infant mortality and neonatal and post-neonatal components according to social vulnerability strata. Municipality of São Paulo, 2006–2015, 2016–2019, and 2006–2019.

Social vulnerability strata	2006–2015			2016–2019			2006–2019		
		
	APV (%)	95%CI	p-value	APV (%)	95%CI	p-value	APV (%)	95%CI	p-value
MRT									
Low	−2.56	−3.53 to −1.59	< 0.001	−0.99	−8.01 to 6.57	0.62	−1.75	−2.54 to −0.95	< 0.001
Medium	−1.77	−3.08 to −0.44	0.015	0.34	−1.53 to 2.25	0.515	−1.06	−1.98 to −0.14	0.028
High	−2.17	−3.00 to −1.34	< 0.001	−0.47	−2.65 to 1.75	0.464	−1.28	−2.14 to −0.42	0.007
Total	−2.25	−3.18 to −1.30	0.001	−0.44	−2.63 to 1.79	0.481	−1.4	−2.36 to −0.42	0.009
Neonatal									
Low	−1.92	−3.08 to −0.74	0.006	2.03	−3.41 to 7.78	0.255	−1.35	−2.19 to −0.49	0.005
Medium	−1.36	−2.43 to −0.28	0.02	−0.34	−7.59 to 7.47	0.863	−0.75	−1.47 to −0.03	0.042
High	−1.61	−2.22 to −1.00	< 0.001	0.49	−2.46 to 3.52	0.556	−0.97	−1.51 to −0.43	0.002
Total	−1.53	−2.25 to −0.80	0.001	0.6	−3.65 to 5.04	0.613	−0.93	−1.64 to −0.21	0.016
Post−neonatal									
Low	−3.99	−5.46 to −2.50	< 0.001	−7.03	−16.65 to 3.70	0.103	−2.58	−3.81 to −1.33	< 0.001
Medium	−2.95	−5.24 to −0.61	0.02	1.74	−7.82 to 12.29	0.53	−1.74	−3.22 to −0.24	0.027
High	−3.83	−6.12 to −1.49	0.006	−2.43	−3.01 to −1.84	0.003	−1.97	−3.52 to −0.39	0.019
Total	−3.52	−5.19 to −1.82	0.001	−2.16	−4.38 to 0.10	0.054	−2.05	−3.30 to −0.78	0.004

IMR: infant mortality rate; APV: annual percentage variation; 95%CI: 95% confidence interval.

When we analyzed infant and neonatal and post-neonatal mortality trends according to social vulnerability strata, we observed that the rates for IMR and its components significantly decreased from 2006 to 2015 in the three strata (p < 0.05). However, the second period (2016 to 2019) showed a stagnant downward trend in the high vulnerability stratum as it significantly declined (APC = −2.43; p = 0.003), except for post-neonatal mortality. However, regarding comparing trends between social vulnerability strata showed no significant differences in all analyzed periods for both IMR and neonatal and post-neonatal mortality ( Table 2 ).

By comparing inequalities in infant mortality and its components between the first and last triennia in the studied period, we observed that, according to RT, infant mortality showed increased inequality and risk of dying in the stratum of high social vulnerability in relation to that of low vulnerability, increasing from 36% in the first triennium to 48% in the last triennium (p = 0.041). In the stratum of medium social vulnerability, the increased risk of dying in the first year of life increased from 19% to 32% (p = 0.007), when compared to the stratum of low social vulnerability. The increases in social inequalities between the two triennia for neonatal and post-neonatal rates failed to reach statistical significance, except for that between the middle and low social vulnerability strata, which increased from 22% to 48% (p = 0.029) ( Table 3 ).

**Table 3:** Differences in infant mortality inequalities according to social vulnerability between the two triennia. São Paulo, 2006–2009 and 2016–2019.

RR ^(*)^	First triennium	Last triennium	p-value
	
	RR (95%CI)	RR (95%CI)	
High/Low SV			
Infant mortality	1.36 (1.27–1.45)	1.48 (1.38–1.59)	0.0414
Neonatal	1.37 (1.26–1.48)	1.44 (1.32–1.57)	0.1919
Post-neonatal	1.34 (1.20–1.50)	1.57 (1.38–1.79)	0.0962
Medium/Low SV			
Infant mortality	1.19 (1.06–1.32)	1.32 (1.27–1.37)	0.0077
Neonatal	1.19 (1.05–1.33)	1.26 (1.12–1.39)	0.0962
Post-neonatal	1.22 (0.81–1.62)	1.48 (1.32–1.64)	0.0293

* Rate Ratio | 95% confidence interval.

## DISCUSSION

Results show a significant drop in infant, neonatal, and post-neonatal mortality rates in MSP, ending in 2015. In the decline period (2006 to 2015), the three IMR strata and post-neonatal rates (compared to neonatal ones) showed a significant reduction. Another important result in this study refers to a significant increase in the social inequality for infant mortality in MSP between the first (2006–2008) and last triennium (2017–2019) in the analyzed period.

Studies conducted in Brazil have also observed recent changes in the trend of infant and childhood mortality. Marinho et al. ^8^ found significant declines in childhood mortality of −3.95% a year from 2001 to 2010 and of −2.35% from 2011 to 2015 and a stabilizing trend from 2016 to 2017 with an insignificant decline of −0.07%. Ferreira et al. ^10^ analyzed IMR in clusters of Brazilian municipalities from 2007 to 2016 and observed a decrease in this indicator from 2007 (16.4/1,000 LB) to 2015 (12.9/1,000 LB), with a slight increase in 2016 (13.4/1,000 LB), with higher concentrations of infant mortality in the Brazilian North and Northeast. Szwarcwald et al. ^9^ found a declining trend in infant mortality from 1990 (47.1) to 2015 (13.5/1,000 LB) but a 3.7% increase in 2016, when compared to 2015, from 10.9 to 11.3 deaths/1,000 LB.

Since we analyzed longer periods than these studies, we found a slight increase in IMR in relation to 2015 and that rates maintained similar values from 2016 to 2019 (11.3 and 11.2 deaths/1,000 LB), although higher than that in 2015.

In this and the aforementioned studies, 2016 had a greater or interrupted IMR decline. That year witnessed a worsened economic crisis and a serious political crisis in Brazil that culminated in the impeachment of its president and the immediate implementation of fiscal austerity measures, including the approval of Constitutional Amendment no. 95/2016 (Spending Ceiling), which reduced budget spending and broadly compromised the health and social protection systems ^7 , 18^ , hitting the population unevenly with increased unemployment and misery rates, preserving the earnings of the richest and penalizing the most vulnerable population ^19^ .

In Brazil, the impact of the economic crisis was evinced from 2014 onward, after a period of expansion from 2004 to 2013, which improved income distribution, reduced poverty ^19^ , and decreased IMR. This study found that the greatest decrease in post-neonatal mortality occurred from 2006 to 2015. Most post-neonatal deaths stem from preventable causes that dispense with high-cost health technologies. We can infer that the expansion of access to primary health care certainly contributed to decreasing IMR in the municipality, but that it insufficiently reduced social inequalities even in that period ^20^ .

Social protection measures and increased health spending could mitigate the effects of economic crises on the population’s health and the mortality of children under one year of age but fiscal austerity policies act in the opposite direction, preventing the application of these protection measures. This context must have expanded the inequalities we observed in infant mortality rates.

MSP, one of the main Latin American economic centers, shows a scenario of great social inequality, which can be represented in several ways. To illustrate this inequality, the proportion of households in *favelas* in relation to the total number of dwellings was higher than 10% in 25 of 96 MSP DA ^11^ in 2020. Moreover, the district of Pinheiros, in Western São Paulo, had a Municipal Human Development Index (MHDI 2010) of 0.942, which was only 0.680 at Parelheiros, at the southern end of city ^21^ .

Another important aspect refers to the fact that while the gross domestic product *per capita* of MSP increased by 118% from 2006 to 2015, the observed growth of this indicator in the same period was only 10% ^22^ , concomitant to the stagnation of the decline in infant mortality and increase in social inequality.

Note that the municipality has a capillary public primary health care network that has grown over the years. From 434 basic health units operating in 2010, MSP had 468 in 2019 and an increase in the number of Family Health Strategy teams, from 928 in 2010 to 1,343 in 2019, with a higher concentration of basic health units and Family Health Strategy teams in the most peripheral areas of the municipality ^23^ . This expansion, however, neither maintained the decline in IMR in recent years nor prevented the expansion of inequality in infant deaths between the most and least vulnerable areas of the municipality.

This study has some limitations inherent to its use of secondary databases and the impossibility of analyzing a longer series since the SMS-SP had no complete databases prior to 2006. The analysis of our results should consider its ecological design, which bases its definition of social vulnerability strata on data from residence areas rather than on an individual basis, which could produce different results. However, this approach can identify areas that need differentiated strategies of actions and signal the need to redirect resources for intervention ^24^ . We should also highlight that, given that the observed rates are already low and inequalities rather small, our option to compare the initial and final triennium of the series proved to be correct since it managed to find the increase in inequality in infant mortality.

The period we analyzed is more recent than that in the literature, enabling us to evince the maintenance of IMR values and the increase in their social inequality. Another aspect we should mention is our use of reliable databases since the SMS-SP maintains a program to improve data quality in Certificates of Live Births and Deaths, reducing incompleteness and inconsistencies.

This study fills an important knowledge gap since we found no recent research in the literature that analyzed the trend and inequality of infant mortality in MSP. Our results show an unfavorable epidemiological scenario since current public policies have failed to continue decreasing infant deaths in the city and have enabled the increase in the socio-spatial inequality of infant mortality. Thus, in addition to directing efforts to expand social security policies, basic sanitation, education, and access to health, including protection measures involving women (from family planning, to prenatal care, childbirth, and puerperium) and monitoring children during their first year of life, it is also necessary to maintain income transfer programs since they can both reduce IMR and inequality ^25 , 26^ and mitigate the effects of income concentration and poverty on infant mortality ^27^ as these determinants seem to have important implications for reducing infant mortality, as per a systematic review by Bugelli et al. ^32^

Results also show the need for continuously monitoring the trend and size of prevalent social inequalities and adopting and reinforcing intervention measures especially directed to the population in areas of medium and high social vulnerability within the perspective of subsidizing policies to advance health equity.

Further studies are needed to develop the understanding of the determinants on the scene and how they interact in a municipality with the characteristics of São Paulo.
