# Supplementary material for: Infant mortality in the municipality of São Paulo: trend and social inequality (2006–2019)
Source: Rev Saude Publica. 2023 Oct 30;57:84. doi: 10.11606/s1518-8787.2023057004791 (PMC10631751; doi:10.11606/s1518-8787.2023057004791)
Supplement: Translation

# Translation: Mortalidade infantil no município de São Paulo: tendência e desigualdade social (2006–2019)

**Authors:** Katia Cristina Bassichetto, Margarida Maria de Azevedo Tenório Lira, Edige Felipe de Sousa Santos, Ivan Arroyave, Samantha Hasegawa Farias, Marilisa Berti de Azevedo Barros

**Affiliations:** I Brazil Faculdade de Ciências Médicas da Santa Casa de São Paulo. Departamento de Saúde Coletiva. São Paulo, SP, Brazil .; II Brazil Pesquisadora autônoma. São Paulo, SP, Brazil .; III Brazil Universidade de São Paulo. Faculdade de Saúde Pública da USP. Departamento de Epidemiologia. São Paulo, SP, Brazil .; IV Colombia Universidad de Antioquia. Facultad Nacional de Salud Pública. Medellín, Colombia .; V Brazil Universidade Estadual de Campinas. Faculdade de Ciências Médicas. Departamento de Saúde Coletiva, Campinas, SP, Brazil .

**Keywords:** Mortalidade Infantil, Fatores de Tempo, Fatores Socioeconômicos, Vulnerabilidade Social

## Abstract

**OBJETIVO:**

Considerando as evidências publicadas sobre o impacto de crises econômicas e da implementação de políticas de austeridade fiscal em vários indicadores de saúde, e a ocorrência recente desses eventos no Brasil, o objetivo deste estudo foi analisar o comportamento da tendência e da desigualdade socioespacial da mortalidade infantil no município de São Paulo, entre 2006 e 2019.

**MÉTODOS:**

Trata-se de estudo ecológico de análise de tendência temporal, desenvolvido no município de São Paulo e em três estratos de áreas de residência, diferenciadas segundo nível de vulnerabilidade social, a partir do Índice Paulista de Vulnerabilidade Social de 2010. Calcularam-se as taxas de mortalidade infantil, neonatal e pós-neonatal para cada um dos estratos de vulnerabilidade social, para cada ano do período e para o primeiro e o último triênios. A tendência temporal foi analisada com o modelo de regressão de Prais-Winsten e a magnitude da desigualdade avaliada pelas razões de taxas.

**RESULTADOS:**

O declínio das taxas de mortalidade infantil e de seus componentes, observado entre 2006 e 2015, que foi mais elevado no estrato de baixa vulnerabilidade social e no período pós-neonatal em comparação ao neonatal, foi interrompido em 2015, com estagnação das taxas no período subsequente (2016–2019). A análise da desigualdade da mortalidade infantil entre os estratos de vulnerabilidade social revelou aumento significativo entre os triênios inicial e final do período analisado; as razões de taxas cresceram de 1,36 para 1,48 entre o estrato de alta em relação ao de baixa vulnerabilidade social e de 1,19 para 1,32 entre o de média e de baixa vulnerabilidade social.

**CONCLUSÕES:**

O estancamento do declínio da taxas de mortalidade infantil em 2015 e o aumento da desigualdade socioespacial observados apontam para a necessidade premente de reformulação das políticas públicas vigentes para reversão desse quadro, visando reduzir a iniquidade presente no risco de morte infantil.

## INTRODUÇÃO

Dados da Organização Mundial da Saúde (OMS) mostram que a taxa de mortalidade infantil (TMI) apresentou declínio de aproximadamente 50%, no período de 2000 a 2018, porém com profunda desigualdade entre regiões e países ^1^ . Esse panorama também foi observado na Região das Américas, onde a TMI caiu 55%, entre 1995 e 2017, mas com grande variação nos valores das taxas entre os países ^2^ .

A literatura tem evidenciado que o processo de aceleração da concentração de renda, a crise econômica e as políticas de austeridade fiscal implementadas têm provocado reflexos negativos em vários indicadores de saúde ^3^ e na mortalidade infantil, como observado em diversos países do mundo ^4^ .

No Brasil, esses mesmos processos também levaram à tendência de piora de alguns indicadores de saúde (fatores de risco e proteção para doenças crônicas não transmissíveis — DCNT —, taxas de mortalidade prematura para DCNT, infantil e materna), com ampliação da desigualdade social entre segmentos sociais da população ^7^ . No que se refere à mortalidade infantil, um estudo brasileiro detectou, em 2016 e 2017, desaceleração da tendência de queda que vinha sendo observada até 2015 ^8^ e outros dois estudos observaram aumento da TMI em 2016, após o declínio verificado até 2015 ^9 , 10^ . Em 2016, registrou-se um aprofundamento da crise econômica e política no Brasil, que atingiu de forma desigual a população do país e afetou os moradores do município de São Paulo (MSP), com elevação da taxa de desemprego e queda da renda, principalmente entre os mais vulnerabilizados ^11^ .

Diante dessa conjuntura e considerando a relevância da TMI, indicador sensível para avaliar as condições de vida e saúde de uma localidade ^12 , 13^ , e como não foram encontrados estudos recentes que tivessem avaliado a tendência das taxas e das desigualdades sociais desse indicador no MSP, o objetivo deste estudo foi analisar a tendência das taxas e da magnitude das desigualdades socioespaciais da mortalidade infantil nesse município, considerando o período de 2006 a 2019.

## MÉTODOS

### Tipo de Estudo

Trata-se de um estudo ecológico com delineamento de séries temporais que utiliza dados de óbitos em menores de 1 ano e de nascidos vivos (NV) do MSP, ocorridos em áreas com diferentes níveis de vulnerabilidade social, entre 2006 e 2019.

Os dados dos óbitos e os de nascidos vivos foram coletados por local de residência e corresponderam ao período entre 1º de janeiro de 2006 e 31 de dezembro de 2019, sendo 2019 considerado o ano mais recente com dados consolidados disponíveis. Optou-se por utilizar 2006 como ano de início da série porque foi somente a partir desse ano que a Secretaria Municipal de Saúde de São Paulo (SMS-SP) passou a realizar o procedimento de retroalimentação dos registros da base de dados consolidada pelo Ministério da Saúde, incorporando na base municipal aqueles que ocorreram em outros municípios.

As bases de dados são provenientes dos Sistemas de Informações sobre Mortalidade (SIM) e sobre Nascidos Vivos (Sinasc). A população de residentes no MSP foi obtida do TabNet da SMS-SP, com projeções realizadas pela Fundação Seade, a partir dos dados do censo de 2010 ^14^ .

### Análise de Desigualdades Socioespaciais

As análises de desigualdades socioespaciais foram realizadas com base no Índice Paulista de Vulnerabilidade Social (IPVS), desenvolvido pela Fundação Seade, que utilizou indicadores socioeconômicos e demográficos para classificar os setores censitários dos municípios do estado de São Paulo em seis grupos de vulnerabilidade social: baixíssima, muito baixa, baixa, média, alta e muito alta. Esse indicador permite identificar e localizar espacialmente as áreas que abrigam os segmentos expostos a diferentes graus de vulnerabilidade social ^15^ .

Para classificar os estratos de vulnerabilidade social do MSP, cada um dos 96 distritos administrativos (DA) do município recebeu um escore de vulnerabilidade, baseado no percentual de setores censitários classificados em cada grupo do IPVS. Em seguida, os DA foram ordenados segundo esses escores, do menor para o maior, e foram classificados em três estratos de vulnerabilidade social (baixa, média e alta) ( Figura 1 ), contendo cada um aproximadamente um terço da população residente no município. O estrato de baixa vulnerabilidade social incluiu 46 DA, o estrato de média incluiu 28 DA e o de alta vulnerabilidade incluiu 22 DA.

**Figura 1:** Classificação dos distritos administrativos do município de São Paulo segundo vulnerabilidade social.

MSP: município de São Paulo; DA: distrito administrativo.

Fonte: adaptado do Índice Paulista de Vulnerabilidade Social (IPVS), Fundação Seade, 2013.

### Análise Estatística

Foram calculadas as TMI (< 1 ano de idade) e as taxas de mortalidade dos componentes neonatal (0–27 dias) e pós-neonatal (28 dias a < 1 ano) por 1.000 nascidos vivos, para os três estratos de vulnerabilidade social e para cada ano do período estudado (2006 a 2019).

Para análises de tendência, foi utilizado o modelo de regressão de Prais-Winsten. As análises de tendência da mortalidade infantil, neonatal e pós-neonatal foram feitas para o período todo (2006–2019) e separadamente para os períodos de 2006 a 2015 e de 2016 a 2019, devido à observação de que o menor valor da TMI havia ocorrido em 2015. Para as séries temporais, considerou-se como variável dependente o logaritmo das taxas; e como variável independente, os anos da série histórica. Investigou-se a existência de autocorrelação serial por meio do teste de Durbin-Watson e foram calculados a variação percentual anual (VPA) das taxas, o coeficiente de regressão (β) e o respectivo intervalo de confiança de 95% (IC95%) e o valor de p, considerando um nível de significância de 95% ^16^ .

Para analisar a magnitude da desigualdade na mortalidade infantil, os anos extremos da série foram agrupados em triênios (2006–2008 e 2017–2019) e calculadas as razões de taxas (RT) entre as áreas de alta e de média vulnerabilidade social em relação às de baixa vulnerabilidade, sendo essa a categoria de referência. Tal procedimento foi realizado para proporcionar maior estabilidade das taxas para as análises.

Para verificar se as RT entre as áreas de alta e de média vulnerabilidade social diferiram entre o primeiro e o último triênio em comparação às de baixa, utilizou-se o teste t de Student, monocaudal à direita, para amostras independentes. Para tomada de decisão estatística, foi considerado o valor de p < 0,05.

A tabulação dos dados, a análise descritiva e os gráficos foram feitos nos programas TabWin (tabulador para Windows), desenvolvido pelo DATASUS, e Microsoft Office Excel 2010. Todas as análises foram realizadas com o programa Stata 15.0 (Stata Corp, LP).

Neste estudo, foram utilizadas bases de dados de óbitos e de nascidos vivos sem identificação, disponibilizadas por meio eletrônico e de forma agregada pela SMS-SP. Portanto, não houve necessidade de submissão ao Comitê de Ética em Pesquisa. O estudo foi realizado em conformidade com a Resolução do Conselho Nacional de Saúde nº 466, de 12 de dezembro de 2012 ^17^ .

## RESULTADOS

No MSP, no período de 2006 a 2019, ocorreram 27.808 óbitos em menores de 1 ano e 2.394.375 em nascidos vivos. Observa-se na Figura 2 que a mortalidade infantil do município declinou de 13,6 óbitos/1.000 nascidos vivos, em 2006, para 10,9/1.000 nascidos vivos, em 2015, quando a redução foi interrompida, e quando a taxa apresentou um valor de 11,2/1.000 nascidos vivos em 2019.

**Figura 2:** Tendência da mortalidade infantil. Município de São Paulo, 2006–2019.

VPA: variação percentual anual; MSP: município de São Paulo.

Quanto à distribuição dos óbitos infantis, observa-se que cerca de 65% deles ocorreram no componente neonatal, em todos os estratos de vulnerabilidade social, no primeiro e no último triênio analisado ( Tabela 1 ).

**Tabela 1:** Valores absolutos (n) e relativos (% e taxas) dos óbitos infantis e seus componentes, segundo estratos de vulnerabilidade social. Município de São Paulo, 2006–2008 e 2017–2019.

Variáveis	2006–2008			2017–2019		
	
	n	%	Taxa	n	%	Taxa
Baixa						
Neonatal	929	65,2	6,88	743	69,6	5,90
Pós-neonatal	495	34,8	3,66	325	30,4	2,58
Infantil	1.424	100	10,53	1.068	100	8,48
Média						
Neonatal	1.427	64,8	8,14	1.234	65,9	7,42
Pós-neonatal	776	35,2	4,43	637	34,1	3,83
Infantil	2.203	100	12,57	1.871	100	11,24
Alta						
Neonatal	1.840	65,6	9,42	1.669	67,7	8,51
Pós-neonatal	964	34,4	4,94	797	32,3	4,06
Infantil	2.804	100	14,35	2.466	100	12,58

A análise da tendência da TMI, considerando o período todo ( Tabela 2 ), revela um declínio significativo de -1,40% ao ano, entretanto, essa queda ocorreu de fato entre 2006 e 2015, com um VPA de -2,25% ao ano (p = 0,001), enquanto entre 2016 e 2019 não se observa declínio significativo (VPA = -0,44; p = 0,481).

**Tabela 2:** Tendência da mortalidade infantil e componentes (neonatal e pós-neonatal), segundo estratos de vulnerabilidade social. Município de São Paulo, 2006–2015, 2016–2019 e 2006–2019.

Variáveis	2006-2015			2016-2019			2006-2019		
		
	VPA (%)	IC95%	Valor de p	VPA (%)	IC95%	Valor de p	VPA (%)	IC95%	Valor de p
TMI									
Baixa	-2,56	-3,53 a -1,59	< 0,001	-0,99	-8,01 a 6,57	0,62	-1,75	-2,54 a -0,95	< 0,001
Média	-1,77	-3,08 a -0,44	0,015	0,34	-1,53 a 2,25	0,515	-1,06	-1,98 a -0,14	0,028
Alta	-2,17	-3,00 a -1,34	< 0,001	-0,47	-2,65 a 1,75	0,464	-1,28	-2,14 a -0,42	0,007
Total	-2,25	-3,18 a -1,30	0,001	-0,44	-2,63 a 1,79	0,481	-1,4	-2,36 a -0,42	0,009
Neonatal									
Baixa	-1,92	-3,08 a -0,74	0,006	2,03	-3,41 a 7,78	0,255	-1,35	-2,19 a -0,49	0,005
Média	-1,36	-2,43 a -0,28	0,02	-0,34	-7,59 a 7,47	0,863	-0,75	-1,47 a -0,03	0,042
Alta	-1,61	-2,22 a -1,00	< 0,001	0,49	-2,46 a 3,52	0,556	-0,97	-1,51 a -0,43	0,002
Total	-1,53	-2,25 a -0,80	0,001	0,6	-3,65 a 5,04	0,613	-0,93	-1,64 a -0,21	0,016
Pós-neonatal									
Baixa	-3,99	-5,46 a -2,50	< 0,001	-7,03	-16,65 a 3,70	0,103	-2,58	-3,81 a -1,33	< 0,001
Média	-2,95	-5,24 a -0,61	0,02	1,74	-7,82 a 12,29	0,53	-1,74	-3,22 a -0,24	0,027
Alta	-3,83	-6,12 a -1,49	0,006	-2,43	-3,01 a -1,84	0,003	-1,97	-3,52 a -0,39	0,019
Total	-3,52	-5,19 a -1,82	0,001	-2,16	-4,38 a 0,10	0,054	-2,05	-3,30 a -0,78	0,004

TMI: taxa de mortalidade infantil; VPA: variação percentual anual; IC95%: intervalo de confiança de 95%.

Ao analisar a tendência da mortalidade infantil e dos componentes neonatal e pós-neonatal, segundo estrato de vulnerabilidade social, observa-se que no período de 2006 a 2015, a redução das taxas ocorreu de forma significativa para os três estratos nas TMI e seus componentes (p < 0,05). Entretanto, no segundo período (2016 a 2019), observou-se estagnação da tendência de queda, exceto para a mortalidade pós-neonatal, no estrato de alta vulnerabilidade, que apresentou declínio significativo (VPA = -2,43; p = 0,003). Todavia, no que se refere à comparação das tendências entre os estratos de vulnerabilidade social, não foram verificadas diferenças significativas em todos os períodos analisados, tanto para a TMI quanto para a mortalidade neonatal e pós-neonatal ( Tabela 2 ).

Comparando as desigualdades na mortalidade infantil e componentes, entre o primeiro e o último triênio do período estudado, observou-se que, segundo as RT, a mortalidade infantil apresentou aumento da desigualdade. O risco de morrer no estrato de alta vulnerabilidade social aumentou de 36% no primeiro triênio para 48% no último triênio (p = 0,041) em comparação ao de baixa vulnerabilidade. No estrato de média vulnerabilidade social, o aumento do risco de morrer no primeiro ano de vida passou de 19% para 32% (p = 0,007), quando comparado ao estrato de baixa vulnerabilidade social. Os aumentos das desigualdades sociais entre os dois triênios, nos componentes neonatal e pós-neonatal, não atingiram significância estatística, com exceção da desigualdade entre o estrato de média e o de baixa vulnerabilidade social, que passou de 22% para 48% (p = 0,029) ( Tabela 3 ).

**Tabela 3:** Diferenças das desigualdades, segundo vulnerabilidade social da mortalidade infantil entre dois triênios. Município de São Paulo, 2006–2009 e 2016–2019.

Razões de taxas (RT)	Primeiro triênio	Último triênio	Valor de p
	
	RT (IC95%)	RT (IC95%)	
Alta / Baixa VS			
Mortalidade Infantil	1,36 (1,27–1,45)	1,48 (1,38–1,59)	0,0414
Neonatal	1,37 (1,26–1,48)	1,44 (1,32–1,57)	0,1919
Pós-neonatal	1,34 (1,20–1,50)	1,57 (1,38–1,79)	0,0962
Média / Baixa VS			
Mortalidade Infantil	1,19 (1,06–1,32)	1,32 (1,27–1,37)	0,0077
Neonatal	1,19 (1,05–1,33)	1,26 (1,12–1,39)	0,0962
Pós-neonatal	1,22 (0,81–1,62)	1,48 (1,32–1,64)	0,0293

VS: vulnerabilidade social; IC95%: intervalo de confiança de 95%.

## DISCUSSÃO

Os resultados deste estudo revelaram que a queda significativa que vinha sendo observada no MSP nas TMI, neonatal e pós-neonatal foi interrompida em 2015. No período de declínio (2006 a 2015), a redução das taxas ocorreu de forma significativa para os três estratos nas TMI e no componente pós-neonatal em comparação ao neonatal. Outro resultado importante do estudo foi a detecção de aumento significativo da desigualdade social da mortalidade infantil no MSP entre o primeiro (2006–2008) e último triênio (2017–2019) do período analisado.

Estudos realizados no Brasil também observaram mudanças recentes na tendência da mortalidade infantil e na infância. Marinho et al. ^8^ verificaram declínios significativos da mortalidade na infância de -3,95% ao ano entre 2001 e 2010 e de -2,35% entre 2011 e 2015, além de tendência de estabilização entre 2016 e 2017, com declínio não significativo de -0,07%. Ferreira et al. ^10^ analisaram a TMI em agrupamentos de municípios brasileiros, no período de 2007 a 2016, e observaram queda desse indicador, entre 2007 (16,4/1.000 nascidos vivos) e 2015 (12,9/1.000 nascidos vivos), com discreto aumento em 2016 (13,4/1.000 nascidos vivos) e maiores concentrações da mortalidade infantil nas regiões Norte e Nordeste do país. Szwarcwald et al. ^9^ constataram tendência de declínio da mortalidade infantil entre os anos de 1990 (47,1) e 2015 (13,5/1.000 nascidos vivos), porém com aumento de 3,7% em 2016 em relação a 2015, passando de 10,9 para 11,3 óbitos/1.000 nascidos vivos.

Tendo em vista que neste estudo a análise avançou por mais tempo do que nas pesquisas mencionadas, foi possível observar um discreto aumento no valor da TMI em relação a 2015 e identificar que as taxas mantiveram valores similares entre 2016 e 2019 (11,3 e 11,2 óbitos/1.000 nascidos vivos), porém superiores ao observado em 2015.

Neste estudo e nas pesquisas citadas, 2016 foi o ano que marcou aumento ou interrupção do declínio da TMI, momento em que ocorria no Brasil um agravamento da crise econômica e política que culminou com o *impeachment* da presidente e a imediata implementação de medidas de austeridade fiscal. Estas incluíram a aprovação da Emenda Constitucional n ^o^ 95/2016 (teto de gastos), que reduziu os gastos orçamentários, comprometendo amplamente o sistema de saúde e o sistema de proteção social ^7 , 18^ . Com isso, a população foi atingida de forma desigual, com aumento das taxas de desemprego e da miséria, preservando os ganhos dos mais ricos e penalizando a população mais vulnerável ^19^ .

No Brasil, o impacto da crise econômica foi evidenciado a partir de 2014, após um período de expansão econômica, entre 2004 e 2013, em que houve maior distribuição de renda e redução da pobreza ^19^ e no qual a TMI vinha declinando. Neste estudo, constatou-se que, entre 2006 e 2015, a maior queda foi observada no período pós-neonatal. Grande parte dos óbitos do componente pós-neonatal se constituem por causas evitáveis e não dependem de tecnologias em saúde de alto custo. Pode-se inferir que a ampliação do acesso à atenção primária à saúde certamente contribuiu para o declínio da TMI no município, mas, mesmo no período de queda, ela não foi suficiente para reduzir as desigualdades sociais ^20^ .

A adoção de medidas de proteção social e o aumento dos gastos com saúde poderiam abrandar os efeitos das crises econômicas na saúde da população e na mortalidade de menores de 1 ano, mas a implementação de políticas de austeridade fiscal atua em sentido contrário, impedindo a aplicação dessas medidas de proteção. Esse contexto deve ter favorecido a ampliação das desigualdades que foram observadas na TMI.

O MSP, um dos principais centros econômicos da América Latina, apresenta um cenário de grande desigualdade social, que pode ser dimensionado de diversas formas. Para ilustrar esse contexto, pode-se verificar, por exemplo, as condições de habitação na cidade. Em 2020, a proporção de domicílios em favelas em relação ao total de habitações foi superior a 10% em 25 dos 96 DA ^11^ e o Índice de Desenvolvimento Humano Municipal (IDHM), em 2010, atingiu 0,942 no distrito de Pinheiros, na zona oeste, e apenas 0,680 em Parelheiros, no extremo sul da cidade ^21^ .

Outro aspecto importante a ser ressaltado é que enquanto o Produto Interno Bruto (PIB) *per capita* do município aumentou 118% entre 2006 e 2015, o crescimento observado desse indicador entre 2016 e 2019 foi de apenas 10% ^22^ , justamente o período de estancamento do declínio da mortalidade infantil e aumento da desigualdade social.

Cabe destacar que o município dispõe de uma rede pública da atenção primária à saúde capilarizada, que vem sendo ampliada ao longo dos anos. De 434 unidades básicas de saúde funcionando em 2010, o MSP passou a ter 468 em 2019, além de ampliar o número de equipes da Estratégia de Saúde da Família, que passou de 928 em 2010 para 1.343 em 2019, com maior concentração de unidades básicas de saúde e de equipes de Estratégia de Saúde da Família nas áreas mais periféricas do município ^23^ . Essa ampliação, entretanto, não possibilitou a manutenção do declínio da TMI nos últimos anos, nem impediu a ampliação da desigualdade nas mortes infantis entre as áreas mais e menos vulneráveis do município.

O estudo apresenta algumas limitações, tanto inerentes ao uso de base de dados secundárias quanto ao fato de não ter sido possível analisar uma série mais longa, uma vez que a SMS-SP não dispunha de bases completas anteriores ao ano de 2006. A análise dos resultados deve considerar que se trata de um estudo ecológico, no qual a definição dos estratos de vulnerabilidade social baseou-se em dados de área de residência, e não de base individual, que poderiam produzir resultados diferentes. Porém, essa abordagem permite identificar áreas que precisam de estratégias diferenciadas de ações e sinalizar a necessidade de redirecionamento de recursos para intervenção ^24^ . Outro aspecto a ser evidenciado é que, visto que as taxas observadas já são baixas e as desigualdades não são tão expressivas, a opção por comparar os triênios inicial e final da série mostrou-se acertada, pois foi possível identificar o aumento da desigualdade na mortalidade infantil.

O período analisado neste estudo avançou em anos mais recentes em relação à literatura, permitindo evidenciar a manutenção dos valores da TMI e o aumento da desigualdade social no indicador. Outro aspecto a ser mencionado é a utilização de bases de dados confiáveis neste estudo, uma vez que a SMS-SP mantém um programa de aprimoramento da qualidade dos dados que constam nas declarações de nascidos vivos e de óbitos, reduzindo incompletudes e inconsistências.

Este estudo supre uma lacuna importante do conhecimento, visto que não foram identificadas pesquisas recentes na literatura que tenham analisado a tendência e a desigualdade da mortalidade infantil no MSP. Os resultados desta análise revelam um cenário epidemiológico desfavorável, tendo em vista que as políticas públicas vigentes não estão conseguindo manter o declínio das mortes infantis no município e estão possibilitando o aumento da desigualdade socioespacial da mortalidade infantil. Sendo assim, além de direcionar esforços para expandir as políticas de seguridade social, de saneamento básico, de educação e de acesso à saúde, incluindo ações de proteção que envolvem as mulheres (planejamento familiar, pré-natal, parto e puerpério) e acompanhamento da criança durante o primeiro ano de vida, também é necessário manter os programas de transferência de renda, já reconhecidos como medidas que contribuem tanto para a redução da TMI quanto para a diminuição da sua desigualdade ^25 , 26^ . Ainda, é preciso mitigar os efeitos da concentração de renda e da pobreza sobre a mortalidade infantil ^27^ , já que todos esses determinantes parecem ter implicações importantes na redução da mortalidade infantil, conforme evidenciado em revisão sistemática realizada por Bugelli et al. ^32^

Os resultados deste estudo revelam também a necessidade de monitoramento contínuo da tendência e da magnitude das desigualdades sociais prevalentes, além da importância da adoção e do reforço de medidas de intervenção direcionadas especialmente à população que vive em áreas de média e alta vulnerabilidade social, na perspectiva de subsidiar políticas que visem avanços na equidade em saúde.

Novos estudos são necessários para aprofundar a compreensão dos determinantes que estão em cena e como eles interagem em um município com as características de São Paulo.
